# Surface modification of PVDF using non-mammalian sources of collagen for enhancement of endothelial cell functionality

**DOI:** 10.1007/s10856-015-5651-8

**Published:** 2016-01-12

**Authors:** Jun Kit Wang, Gordon Minru Xiong, Baiwen Luo, Chee Chong Choo, Shaojun Yuan, Nguan Soon Tan, Cleo Choong

**Affiliations:** Residues and Resource Reclamation Centre (R3C), Nanyang Environment and Water Research Institute (NEWRI), Nanyang Technological University, 1 Cleantech Loop, Singapore, 637141 Singapore; Interdisciplinary Graduate School, Nanyang Technological University, 50 Nanyang Avenue, Singapore, 639798 Singapore; School of Materials Science and Engineering, Nanyang Technological University, 50 Nanyang Avenue, Singapore, 639798 Singapore; School of Biological Sciences, Nanyang Technological University, 60 Nanyang Avenue, Singapore, 637551 Singapore; College of Chemical Engineering, Sichuan University, 19 Wangjiang Road, Wuhou, Chengdu, Sichuan China; Institute of Molecular and Cell Biology, 61 Biopolis Drive, Proteos, A*STAR, Singapore, 138673 Singapore; KK Research Centre, KK Women’s and Children Hospital, 100 Bukit Timah Road, Singapore, 229899 Singapore

## Abstract

Although polyvinylidene fluoride (PVDF) is non-toxic and stable in vivo, its hydrophobic surface has limited its bio-applications due to poor cell-material interaction and thrombus formation when used in blood contacting devices. In this study, surface modification of PVDF using naturally derived non-mammalian collagen was accomplished via direct surface-initiated atom transfer radical polymerisation (SI-ATRP) to enhance its cytocompatibility and hemocompatibility. Results showed that Type I collagen was successfully extracted from fish scales and bullfrog skin. The covalent immobilisation of fish scale-derived collagen (FSCOL) and bullfrog skin-derived collagen (BFCOL) onto the PVDF surface improves the attachment and proliferation of human umbilical vein endothelial cells (HUVECs). Furthermore, both FSCOL and BFCOL had comparable anti-thrombogenic profiles to that of commercially available bovine collagen (BVCOL). Also, cell surface expression of the leukocyte adhesion molecule was lower on HUVECs cultured on non-mammalian collagen surfaces than on BVCOL, which is an indication of lower pro-inflammatory response. Overall, results from this study demonstrated that non-mammalian sources of collagen could be used to confer bioactivity to PVDF, with comparable cell-material interactions and hemocompatibility to BVCOL. Additionally, higher expression levels of Type IV collagen in HUVECs cultured on FSCOL and BFCOL were observed as compared to BVCOL, which is an indication that the non-mammalian sources of collagen led to a better pro-angiogenic properties, thus making them suitable for blood contacting applications.

**Electronic supplementary material:**

The online version of this article (doi:10.1007/s10856-015-5651-8) contains supplementary material, which is available to authorized users.

## Introduction

Due to its excellent thermal stability, high chemical and degradation resistance and favorable mechanical properties, polyvinylidene fluoride (PVDF), is both biostable and non-toxic in vivo [1]. However, the lack of surface functionalisable groups on PVDF for protein-ligand interaction leads to poor cell adhesion and proliferation [2]. Thus, surface modification is required in order to enhance cell-material interactions on PVDF. Improved biocompatibility of PVDF can be achieved by introducing bioactive molecules via physisorption or chemical bonding [3], however, physically-adsorbed molecules tend to detach upon exposure to heat or solvent, since they are held in place by weak Van der Waals forces [4]. As such, the alternative approach of using chemical bonds is preferred.

Surface-initiated atom transfer radical polymerisation (SI-ATRP) is an example of a versatile and robust chemical surface modification process that can be used for a large variety of monomers and functional groups. This method relies on the reversible redox activation of dormant alkyl halide terminated polymer chain end by halogen transfer to a transition metal complex. SI-ATRP has previously been used to effectively improve surface hydrophilicity and anti-fouling properties of PVDF by grafting of hydrophilic polymers from the secondary fluorine atoms of the PVDF backbone [5]. The pendant reactive groups on the side chain of polymer brushes, including hydroxyl, amino, carboxyl and epoxy group, served as the anchoring sites for the binding of biomolecules and proteins, such as collagen, to promote cell-material interaction [1].

Collagen, as a natural material, has excellent biocompatibility, low antigenicity and is able to biodegrade into physiologically non-toxic products [6]. Thus, collagen has wide applications in tissue engineering either on its own, or in combination with other biomaterials as hybrid materials for the fabrication of porous scaffolds for bone graft, skin substitute, drug delivery, wound dressings and artificial blood vessel [7]. Currently, the main commercial source of collagen is acid-solubilised collagen (ASC), which is often derived from non-human sources, such as bovine or porcine dermis and bone [6]. However, the clinical applications of these materials have been limited due to the outbreaks of bovine spongiform encephalopathy (BSE), transmissible spongiform encephalopathy (TSE) and foot and mouth disease (FMD) in cattle and pigs. Furthermore, collagen derived from porcine material may have religious restrictions in certain countries [6, 8]. In addition, although recombinant collagen has been developed to minimise the diseases transmission associated with animals, this process requires complex processing steps involving a large number of enzymes [9]. Therefore, an alternative source of collagen is highly desirable.

Although both fish and bullfrog serve as sources of meat for human consumption here in Asia, the scales from the fish and the skin from the bullfrog are commonly treated as waste material from farm processing as they are inedible [10]. However, these waste products can potentially serve as low cost, alternative sources of protein to replace mammalian collagen, since no risk of animal-related disease transmission has been associated with both fish scale-derived collagen (FSCOL) and bullfrog skin-derived collagen (BFCOL) [6, 10]. In fact, the in vivo biocompatibility of FSCOL has recently been demonstrated in its clinical application as a scaffold to cultivate corneal cells for corneal regeneration [11]. Collagen derived from bullfrog skin has also been used as a collagen film for drug delivery using a model protein, bovine serum albumin (BSA) [12]. However, at present, limited studies have been carried out to compare between different sources of non-mammalian collagen and a detailed study involving the conjugating of these extracted collagens to PVDF surfaces via SI-ATRP method to compare their efficacy for improving biocompatibility and cell proliferation has not yet been carried out. Such investigations will allow for more wide-scale application of these non-mammalian sources of collagens in biomedical research applications.

In the current study, the efficiency of the acid solubilisation method for extracting collagen from fish scales and bullfrog skin was investigated and the isolated collagen material was further characterised by attenuated total reflection-Fourier transform infrared spectroscopy (ATR-FTIR) and sodium dodecyl sulfate polyacrylamide gel electrophoresis (SDS-PAGE) analyses. The acid solubilisation method was used instead of pepsin solubilised treatment to prevent the conversion of collagen into monomeric subunits by pepsin. This conversion would otherwise have caused a loss of telepeptides and crosslinking sites, which would have affected the effectiveness of the collagen crosslinking mechanism [13]. Subsequently, the SI-ATRP method was used to modify the hydrophobic PVDF surface with the different sources of extracted collagen. Commercially available Type I collagen from bovine Achilles tendon (BVCOL) was used as a control. The collagen-enriched PVDF films were characterised by ATR-FTIR. In parallel, in vitro cellular studies were carried out to assess the biocompatibility and cell-material interaction of the different sources of collagen conjugated onto the functionalised PVDF by observing the proliferation profile and the extent of endothelialisation of human umbilical vein endothelial cells (HUVECs). In addition, the hemocompatibility of the endothelialised PVDF films was evaluated by observing the platelet activation, and the inflammatory response of different sources of collagen at the material-tissue interface was investigated by observing the expression levels of leukocyte adhesion markers ICAM-1 and VCAM-1.

## Experimental section

### Materials

Polyvinylidenefluoride (PVDF) sheets were purchased from Goodfellow Cambridge Ltd. (Huntingdon, Cambridgeshire, UK). Scales from giant snakehead (*Channa Micropeltes*) and skin tissue from American bullfrog (*RanaCastebeiana*) were kindly provided by KhaiSeng Trading & Fish Farm Pte Ltd, Singapore. Acetic acid (99.8–100.5 %), lyophilised collagen from bovine Achilles tendon, sodium hydroxide (NaOH, ≥ 98 %), sodium chloride (NaCl, ≥ 99 %), 2-hydroxyethyl methacrylate (HEMA, 97 %), copper (I) bromide (CuBr, 98 %), copper (II) bromide (CuBr_2_, 99 %), 1,1,4,7,10,10-hexamethyltriethylenetetramine (HMTETA, 97 %), anhydrous dimethyl sulfoxide (DMSO, ≥ 99.5 %), 1,1′-carbonyldiimidazole (CDI, reagent grade), tetrahydrofuran (THF, ≥ 99.9 %), fluorescein diacetate, primer sets, bovine serum albumin (BSA, ≥ 98 %), adenosine-5′-diphosphate (ADP, ≥ 95 %), 3,3′,5,5′-Tetramethylbenzidine (TMB, ≥ 99 %) and sulfuric acid (99.999 %) were obtained from Sigma-Aldrich Chemical CO. (St. Louis, MO, USA) and were used without further purification. SnakeSkin^®^ dialysis tubing (10 K MWCO) and P-selectin (CD62P, Clone AK-6) were purchased from Thermo Scientific-Pierce (Rockford, IL, USA). Disodium ethylenediaminetetraacetatedehydrate (EDTA, ≥ 99 %), Bio-Safe™ Coomassie Brilliant Blue R-250, 10 % Tween 20 and SsoAdvanced™ SYBR^®^ Green Supermix were obtained from Bio-Rad (Hercules, CA, USA). Human umbilical vein endothelial cells (HUVECs, EndoGRO™, SCCE001) and EndoGRO-LS Complete Media Kit (SCME001) were purchased from Merck Millipore (Darmstadt, Hesse, Germany). PrestoBlue^®^ Cell Viability Reagent (Molecular Probes^®^), SuperScript^®^ III First-Strand Synthesis SuperMix (Invitrogen™) and Alexa Fluor^®^ 488 goat anti-mouse IgG (H + L) antibody were obtained from Life Technologies (Singapore). RNeasy^®^ Mini Kit was obtained from Qiagen (Hilden, North Rhine-Westphalia, Germany). Purified mouse IgG1 κ isotype control antibody, purified anti-human CD54 (ICAM-1) and purified anti-human CD106 (VCAM-1) were obtained from Biolegend^®^ (San Diego, CA, USA). Anti-mouse IgG (H + L) antibody (Human Serum Adsorbed and Peroxidase Labeled) was obtained from Kirkegaard & Perry Laboratories, Inc. KPL (Gaithersburg, MD, USA). Recombinant human tumour necrosis factor alpha (TNFα) was obtained from R&D Systems™ (Minneapolis, MN, USA).

### Isolation and purification of collagen from fish scales and bullfrog skin

ASC was extracted from fish scales and bullfrog skin following a previously established method of acid solubilisation and followed neutral salt precipitation [14, 15]. All the extraction steps were performed at 4 °C unless otherwise specified. Briefly, for the isolation of collagen from fish scales, the fish scales were washed thoroughly with distilled water for several times to remove impurities. Subsequently, the scales were treated with 0.5 M NaOH of solid/solvent ratio of 1:10 (w/v) to remove non-collagenous protein and pigments for 48 h while changing the solution every 24 h. After 48 h of alkaline treatment, calcium phosphate compounds were removed from the scales prior to acid extraction. Decalcification was performed by stirring the scales in 0.5 M EDTA at pH 7.7 for 48 h with changes in the solution every 24 h to allow for more effective decalcification of the scales [15]. The scales were rinsed with distilled water and extraction was allowed to proceed in 0.5 M acetic acid for 48 h. Collagen was salted out by transferring the viscous extract into a clean flask where the extracted filtrate was mixed with NaCl to a final concentration of 0.9 M and left undisturbed to induce salting out of collagen. The fibrillar suspension was then centrifuged at 4 °C, 10,000×*g* for 1 h and the supernatant was removed. The protein pellet was subsequently reconstituted in a small amount of 0.5 M acetic acid and dialysed using SnakeSkin^®^ dialysis tubing against 0.1 M acetic acid, followed by distilled water for 24 h each. The purified collagen solution was lyophilised and kept at 4 °C for further use [14]. The collagen extraction process for bullfrog skin was similar to that for fish scales, except that the demineralisation step was not required.

### Characterisation of collagen

The collagen fibres in the fish scale and bullfrog skin were imaged prior to the extraction process using a scanning electron microscope (SEM; JEOL Co., Tokyo, Japan). The samples were sputtered with a thin layer of gold using SPI Module sputter coater (SPI Supplies Inc., West Chester, PA, USA) and imaged under an accelerating potential of 5 kV. The chemical composition of the extracted collagen was characterised using Spectrum GX Fourier transform infrared spectrometer (FTIR, Perkin Elmer Inc. Waltham, MA, USA) equipped with a smart performer accessory (i.e. germanium (Ge) crystal with an incident angle of 45°), and a sampling area of 2 mm with attenuated total reflection (ATR) mode to identify the different functional groups of the collagen. BVCOL was used as control. The extracted collagen was pressed onto the Ge crystal and the resulting spectra were collected at a resolution of 4 cm^−1^ in the range of 4000–650 cm^−1^ over 32 scans. The amino acid composition of the FSCOL and BFCOL was characterised qualitatively and quantitatively using the Hitachi amino acid analyser L-8900 (Hitachi, Tokyo, Japan) with BVCOL as a control. Sodium dodecyl sulfate polyacrylamide gel electrophoresis (SDS-PAGE) of the isolated collagen was performed following the method established by Laemmli [16]. 10 µg of lyophilised collagen was dissolved in 1× SDS Loading Dye, heated at 95 °C for 5 min and resolved in 10 % polyacrylamide gel; migration at 0.2 A for 1 h 45 min. After electrophoresis, the gel was stained with Bio-Safe™ Coomassie Brilliant Blue R-250 for visualisation of the protein bands. The protein bands were visualised using enhanced chemiluminescent substrate (ECL) on photographic film. BVCOL was used as markers of alpha- (α), beta- (β) and gamma- (γ) chains mobilities [17, 18].

### Surface functionalisation of PVDF films via direct surface-initiated ATRP of HEMA

**Scheme 1:**
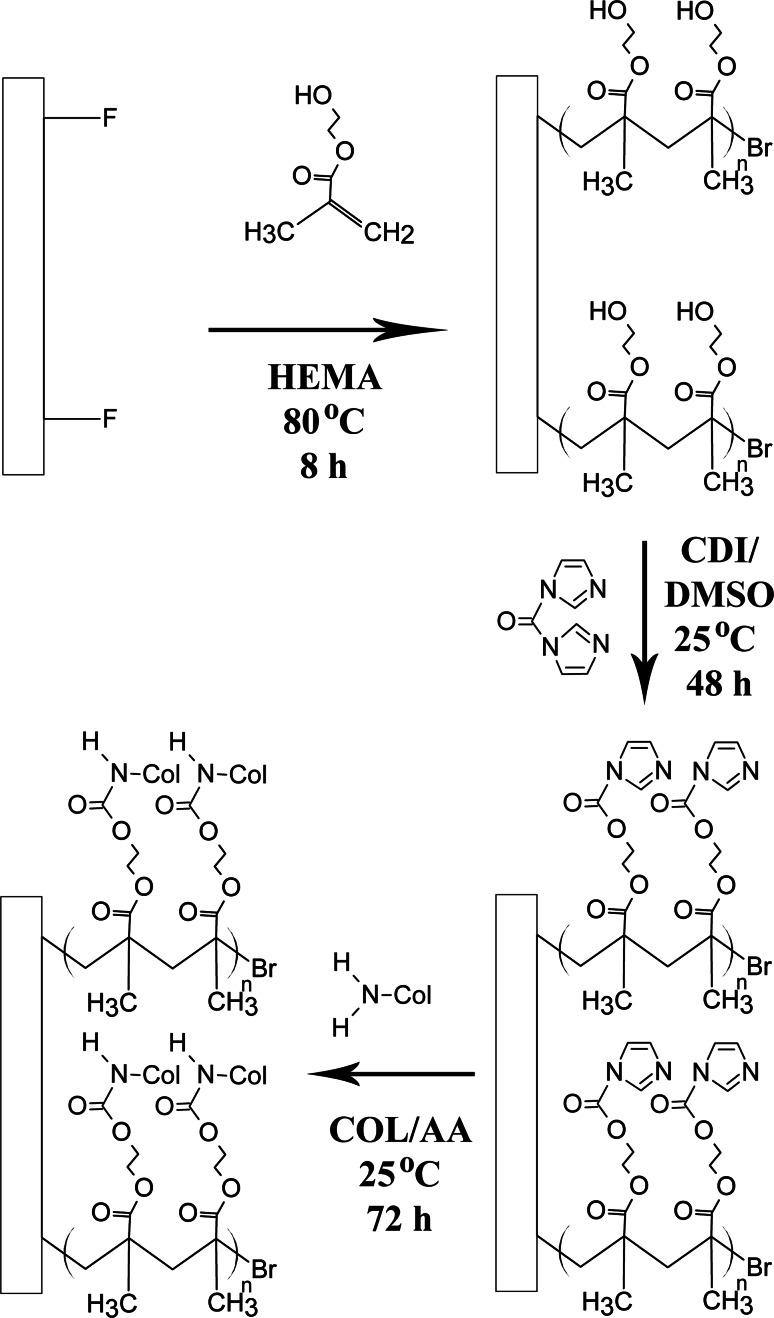
Reaction path used for surface grafting of collagen from PVDF by direct SI-ATRP

The hydrophilic pHEMA brushes were first grafted from the PVDF surface via direct SI-ATRP using the secondary fluorine atoms as initiating sites. The overall reaction pathway for the surface functionalisation of PVDF with collagen using the SI-ATRP method is illustrated in Scheme 1. Briefly, the PVDF films were cut into circular-shaped specimens of 1.5 cm in diameter each. The films were soaked in acetone, ethanol and deionised water for 5 min each time to remove grease or organic contaminants before being dried in a vacuum oven at 40 °C. A typical solution polymerisation process with a molar ratio of [HEMA (monomer)]:[CuBr (catalyst)]:[CuBr_2_ (deactivator)]:[HMTETA (ligand)] at 100.0:1.0:0.2:2.0 in deionised water was carried out as follows. The clean PVDF films were added into a 50 mL round bottom flask containing 5 mL of deionised water and 1.5 mL of HEMA (12.37 mmol). After the reaction mixture was degassed with pure argon for 30 min, 17.7 mg of CuBr (0.12 mmol), 5.5 mg of CuBr_2_ (0.025 mmol) and 68 µL of HMTETA (0.25 mmol) were added into the reaction mixture. The reaction tube was sealed and placed in an 80 °C oil bath for 8 h. At the end of reaction period, PVDF films were removed from the flask and washed with copious amount of ethanol and deionised water to remove the physically-adsorbed reactants, if any. The resultant pHEMA-grafted PVDF films (defined as PVDF-*g*-pHEMA) were dried in vacuum oven at 40 °C and stored at room temperature in a drying cabinet AD-080 (Digi-Cabi, Singapore) prior to being used.

### Collagen immobilisation onto the functionalised PVDF films

To immobilise the extracted collagen onto the PVDF films, the PVDF-*g*-pHEMA films were immersed in 5 mL of DMSO containing 0.5 g of CDI to activate the terminal hydroxyl groups (–OH) on the side chains of the pHEMA brushes. The reaction was allowed to proceed at room temperature for 48 h to give rise to the PVDF-*g*-pHEMA-CDI surface. After their removal from the reaction mixture, the resulting substrates were rinsed with copious amounts of THF and deionised water, and dried in a 37 °C oven for 24 h. The dried films were subsequently immersed in 3 mg/mL of collagen solution dissolved in 0.5 M acetic acid, and mildly stirred at room temperature for 72 h. The film samples were taken out from the flask after reaction and washed with ethanol, followed by deionised water, before drying in vacuum oven preset at 40 °C for further usage. The successful grafting of the polymer brushes via SI-ATRP and the conjugation of collagen were confirmed using ATR-FTIR analyses using similar procedures described in Sect. 2.3, since different functional groups on the films’ surface at the end of each reaction step could be identified using this method.

### In vitro endothelial cell culture

Cellular studies involving HUVECs were performed using previously established protocols [19]. Briefly, HUVECs were seeded and expanded in tissue culture flasks using defined, low-serum EndoGRO-LS endothelial cell culture medium supplemented with 100 U/mL penicillin and 100 g/mL streptomycin. The cells were maintained in a 5 % CO_2_ environment at 37 °C with saturated humidity, and the culture medium was replaced every alternate day. The cells were subcultured upon reaching 80 % confluency first by washing in 1× phosphate buffered saline (PBS), and followed by detaching them from culture flask surfaces using 0.25 % Trypsin–EDTA. HUVECs between passages 4–6 were used for subsequent experiments.

### Cell proliferation and viability studies

The PVDF films were sterilised with ethylene oxide (EtO) for 12 h prior to cell seeding with an initial density of 5 × 10^4^ cells per film in a 12-well plate. The HUVECs were cultured at 37 °C, 5 % CO_2_ in a saturated humidity. Cell proliferation was determined on days 1, 3, 5 and 7 using the PrestoBlue^®^ cell viability reagent according to the manufacturer’s instructions [19]. Briefly, at the end of each incubation period, culture medium was removed and 0.5 mL of 10 % PrestoBlue^®^ solution (diluted in culture medium) was added to the wells. The plates were then incubated in a 5 % CO_2_ atmosphere at 37 °C for 1 h. The fluorescence analysis using an excitation wavelength of 560 nm and an emission wavelength of 590 nm was carried out with a SpectraMax M2 microplate reader (Molecular Devices, Sunnyvale, CA, USA). Cell numbers were calculated using a standard curve correlating known cell numbers with fluorescence.

After incubation for 7 days, the confluency and viability of the HUVECs covering the collagen-enriched PVDF films was observed using fluorescence-based chemicals, as a measure of the extent of endothelialisation on the collagen-enriched PVDF films. Fluorescein diacetate (FDA) was used to assess the viable live cells (green fluorescence). Briefly, culture medium was removed from each well and each sample was washed with 1× PBS for three times. The working solution containing 20 µM FDA was then added directly to each sample. After incubation at room temperature for 5 min, the surfaces were observed using a Zeiss Axio Observer.Z1 inverted fluorescence microscope fitted with a camera (Carl Zeiss MicroImaging GmbH, Göttingen, Germany) at an excitation of 490 nm and an emission of 515 nm on each of the surfaces.

### RNA isolation and gene expression using real-time polymerase chain reaction (qPCR)

The phenotypes of the cells seeded on the collagen-enriched PVDF films were examined by comparing mRNA expression for pro-thrombotic factors [von Willebrand factor (vWF) and plasminogen activator inhibitor-1 (PAI-1)], anti-thrombotic factors [tissue plasminogen activator (tPA) and endothelial nitric oxide synthase (eNOS)] and Type IV collagen. RNA was isolated from HUVECs on the collagen-enriched PVDF films after 7 days of growth using RNeasy^®^ Mini Kit according to manufacturer’s directions. Yield of the extracted RNA was quantified by spectrophotometric measurement using NanoDrop™ 2000 (Thermo Scientific, Wilmington, DE, USA) and 100 ng of total RNA was taken for reverse transcription into cDNA using the SuperScript^®^ III First-Strand Synthesis SuperMix. The primer sets used (Table 1) were obtained from previously published reports and synthesised by OLIGO Sigma [20, 21].

**Table 1:** Sequences of primers for quantitative qPCR

Transcripts	Primer sequence (5′ to 3′)
vWF	S: CACCATTCAGCTAAGAGGAGG
	A: GCCCTGGCAGTAGTGGATA
PAI-1	S: TTGGTGAAGGGTCTGCTGTG
	A: GGCTCCTTTCCCAAGCAAGT
tPA	S: ATGGGAAGACATGAATGCAC
	A: GAAAGGGGAAGGAGACTTGA
eNOS	S: AGCTGTGCTGGCATACAGGA
	A: ATGGTAACATCGCCGCAGAC
Collagen IV	S: CCTGGCTTGAAAGGTGATAAG
	A: CCCGCTATCCCTTGATCTC
GAPDH	S: CCCCTTCATTGACCTCAACTACA
	A: TTGCTGATGATCTTGAGGCTGT

qPCR was performed on a Bio-Rad C1000™ thermal cycler (Bio-Rad, Hercules, CA, USA). Reactions were carried out in a total volume of 20 μL containing 10 μL SsoAdvanced™ SYBR^®^ Green supermix, 2 μL template cDNA, 200 nM forward primer, 200 nM reverse primer, where the remaining amount is filled with RNase/DNase-free water, at an annealing temperature of 58 °C using previously established conditions [19]. GAPDH was used as the internal housekeeping control for the normalisation of initial cDNA loading amounts across different samples. The threshold cycle (Ct) values obtained from qPCR were used in the calculation of relative mRNA expression by the ∆∆Ct method while all values obtained were compared and normalised against the tissue culture plastic (TCPs) control.

### Platelet activation study

Citrated whole blood was collected from healthy human volunteers using procedures approved by the Nanyang Technological University Institutional Review Board (IRB-2014-08-002). Platelet activation on the different sources of collagen with an endothelialised surface was investigated through the detection of P-selectin (CD62P) expressed on freshly-derived platelets using previously established protocols [22]. Briefly, 500 μL of fresh human platelet rich plasma (PRP) was incubated with the endothelialised collagen surfaces at 37 °C for 2 h. At the end of the incubation, the surfaces were washed thoroughly three times with copious amounts of 1× PBS solution, followed by the addition of 1 mL of staining buffer (5 w/v% BSA in 1× PBS) for blocking of non-specific antibody binding sites for 15 min. Following that, 5 μL of anti-CD62P IgG1 (1:200 v/v%) was added to each sample, followed by incubation at 37 °C for 1 h. Non-activated platelets incubated with non-staining antibody (IgG1 isotype control, 1:200 v/v%) were used as the negative control for the staining, while adenosine diphosphate (ADP)-activated platelets (treatment with 30 µM ADP, 2 h) incubated with anti-CD62P antibody was used as the positive control for the assay. The samples were being washed three times with PBS-T (5 w/v% BSA in 1× PBS with 0.5 v/v% Tween-20) before incubation with 5 μL of horseradish peroxidase-conjugated sheep anti-mouse polycolonal antibody at 1:200 v/v% and at 37 °C for 1 h. Subsequently, the samples were washed three times with PBS-T, followed by the addition of 200 μL of 3,3′,5,5′-tetramethylbenzidine (TMB) chromogenic solution for 10 min. The colour reaction was stopped by addition of 400 μL of 1 M H_2_SO_4_, and the optical densities (OD) were measured at 450 nm using a SpectraMax M2 microplate reader (Molecular Devices, Sunnyvale, CA, USA) [19, 22].

### Quantitation of cell adhesion molecules ICAM-1 and VCAM-1 by flow cytometry

The material-induced inflammatory response of different sources of collagen was further investigated by looking at the interactions at the material-tissue interface through the expression of leukocyte adhesion molecules by HUVECs seeded on the collagen. On the HUVECs, intracellular adhesion molecule (ICAM-1) and vascular cell adhesion molecule (VCAM-1) are the cytokine-regulated cell surface molecules that mediate leukocyte migration and adhesion to the endothelium as part of the responses elicited during vascular injury or inflammation. Thus, the expression levels of ICAM-1 and VCAM-1 are indicators of HUVECs activation during pro-inflammatory states [23, 24]. HUVECs cultured on different sources of collagen were trypsinised and washed with 1× PBS before incubation with staining buffer (5 w/v% BSA in 1× PBS) for blocking of non-specific antibody binding sites for 15 min on ice. ICAM-1 or VCAM-1 mouse IgG anti-human antibodies (1:200 v/v%) were then added to the HUVECs in staining buffer on ice and incubated for 45 min. HUVECs stained with mouse IgG isotype control antibody were used as the negative control, while TNFα-stimulated HUVECs (treatment with 100 ng/mL TNFα, 16 h) was used as the positive control for the assay. The HUVECs were then washed twice with PBS-T by centrifugation, following which the HUVECs were stained with goat Alexa Fluor-488-conjugated anti-mouse IgG (H + L) antibody (1:200 v/v%) for 20 min on ice. The HUVECs were washed twice with PBS-T before the surface expression of ICAM-1 and VCAM-1 was determined using the LSRFortessa™ X-20 flow cytometer (BD Biosciences, San Jose, CA, USA) [23, 25].

### Statistical analysis

The quantitative results in this study were expressed as mean ± standard deviation (SD). All experiments were carried out in triplicate (*n* = 3) unless otherwise specified and statistical significance was determined using Kruskal–Wallis nonparametric one-way analysis of variance and Mann–Whitney *U* test. The differences were considered statistically significant when *P* < 0.05.

## Results

### Characterisation and confirmation of extraction of Type I collagen

**Fig. 1:**
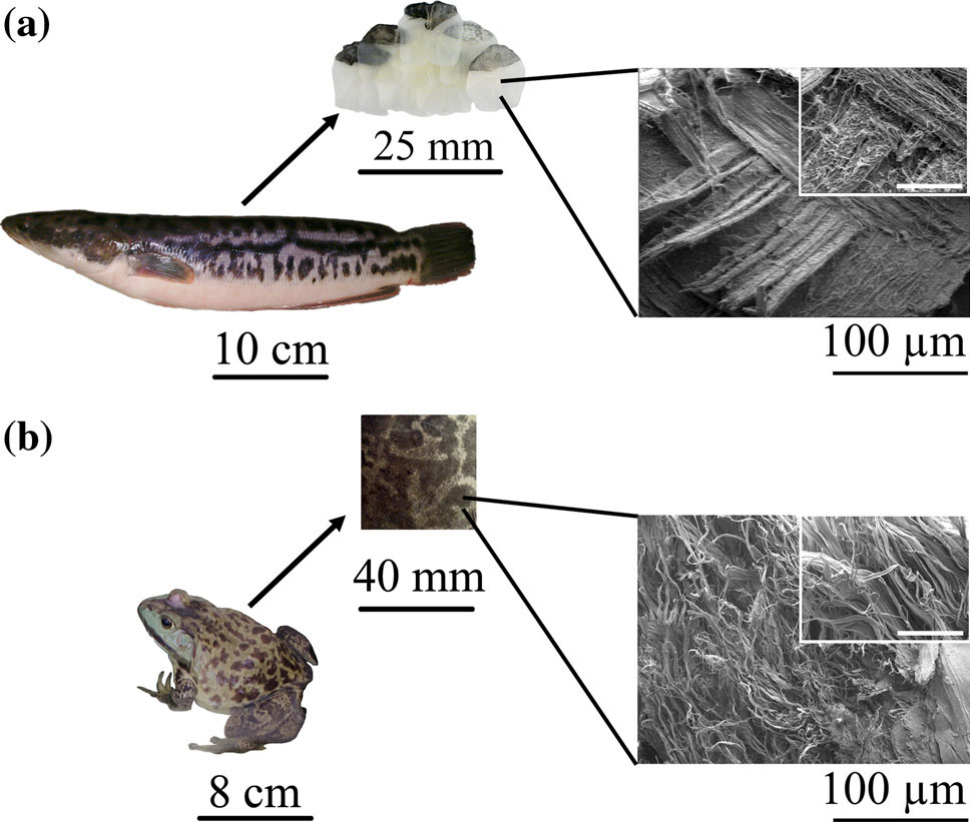
Extraction of collagen from food processing waste material in the form of **a** Snakehead scales and **b** Bullfrog skin. The SEM images show the presence of collagen fibres of different thicknesses and orientation. (*Inset*: *Scale bar* = 50 µm)

**Fig. 2:**
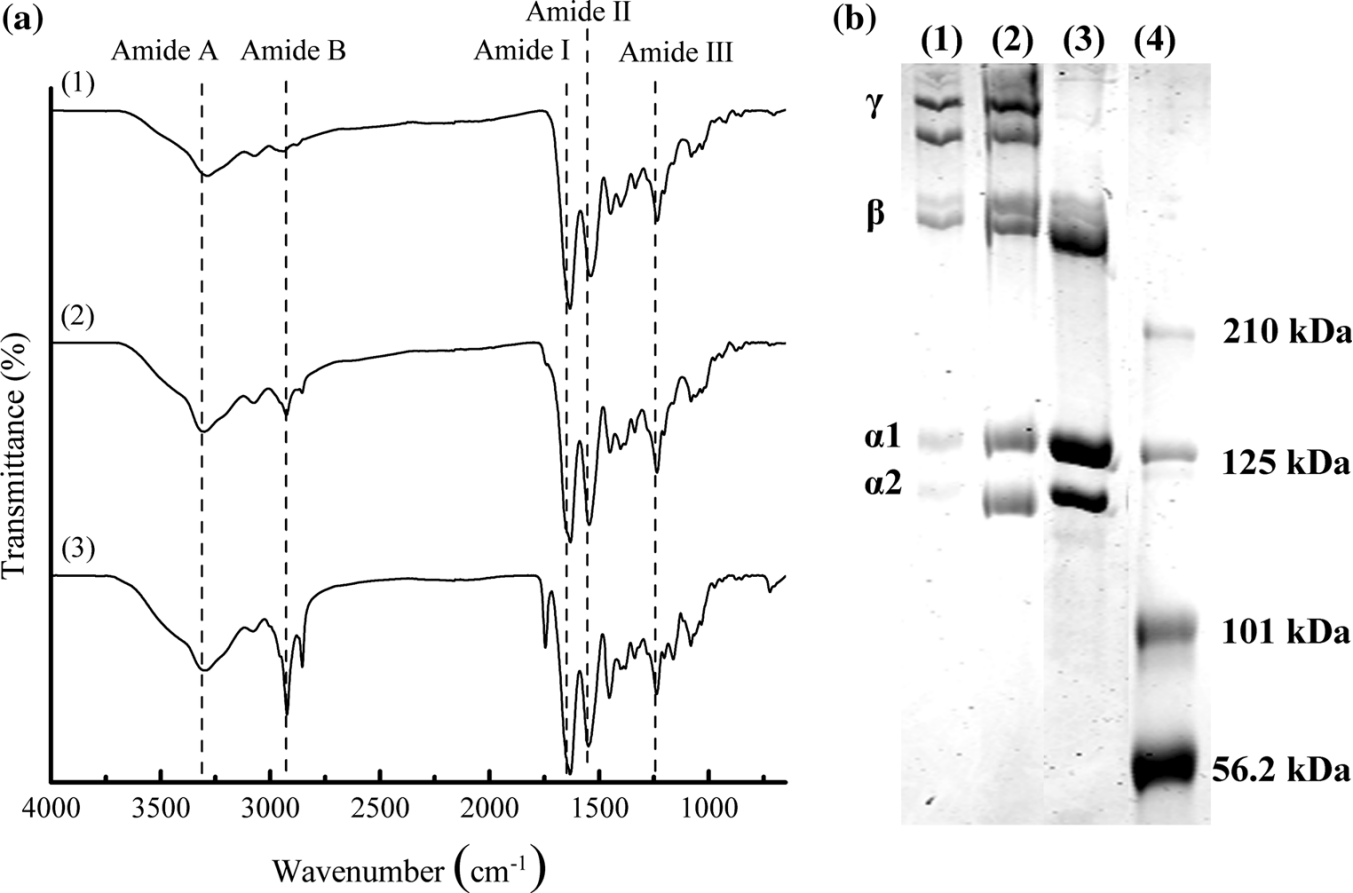
Characterisation of collagen showing **a** ATR-FTIR spectra of the characteristic amide peaks of collagen (*Dotted lines*) and **b** SDS-PAGE analysis showing the protein expression levels of Type I collagen. (*1*) FSCOL, (*2*) BFCOL, (*3*) BVCOL and (*4*) protein ladder as marker

SEM analysis showed the presence of collagen fibres in both fish scale and bullfrog skin prior to the extraction process (Fig. 1). The fish scale of giant snakehead consisted of tightly packed co-aligned collagen fibres with diameters of 0.20–0.41 µm. As the result of the perpendicular alignment of the collagen fibres between the adjacent lamellae planes, highly ordered plywood structures were observed, similar to those reported by others showing the unique arrangement in teleost fish [26, 27]. Meanwhile, a random distribution of collagen fibres with thicker diameters of 1.21–3.63 µm was observed for the bullfrog skin collagen. The thick bundles of collagen fibres had a flat, tape-like shape that divided into fine bundles that ran in various directions, intersecting one another to form a loose meshwork structure. After the extraction process, ATR-FTIR analysis showed the presence of characteristic amide peaks representing amide A, B, I, II and III for the FSCOL and BFCOL samples, which were also found in BVCOL (Fig. 2a). The ATR-FTIR band wavenumber for amide A is located at about 3300 cm^−1^ while amide B is at 2920 cm^−1^. The presence of amide I is indicated by the appearance of the C=O bond at about 1630 cm^−1^, amide II is contributed by the N–H bond (1530 cm^−1^) and amide III is typically detected through complex bands from a mixture of several coordinate displacements (around 1200 cm^−1^) [28]. The presence of the peak at 1450 cm^−1^ for all the FSCOL, BFCOL and BVCOL indicated the presence of the collagen triple helical structure where the ratio of the absorbance peak intensity of amide III to 1450 cm^−1^ is a measurement of the relative amount of the helical structure preserved in the collagen [29]. The ratio of the absorbance peak intensities of amide III and 1450 cm^−1^ for FSCOL, BFCOL and BVCOL were all around 1.0, thus indicating that the helical structures of all the samples were similar to each other and to ASC from other sources [28].

The amino acid compositions of the different sources of collagen are shown in Table 2, where glycine was found to be the most abundant amino acid, which is typical for collagen [6]. Amongst the different sources of collagen, FSCOL contained higher amounts of threonine, alanine and histidine and lower amounts of arginine, aspartic acid, leucine and proline, while BFCOL contained higher amounts of arginine, glycine and aspartic acid as well as serine, lysine and proline as compared to BVCOL. In general, arginine, serine and lysine are amino acid residues involved in post-translational modifications of proteins such as acetylation, methylation and phosphorylation [30], while hydroxylation of proline has been proposed to be important for folding and stabilisation of the collagen triple helix [31]. Hence, the differences in the amino acid composition could potentially lead to different collagen-material interactions.

**Table 2:** Amino acid composition of FSCOL and BFCOL with BVCOL as reference

Types of amino acid	FSCOL (residues/1000)	BFCOL (residues/1000)	BVCOL (residues/1000)
Aspartic acid	41.40	49.29	48.45
Threonine	26.01	20.88	18.41
Serine	36.77	50.10	33.93
Glutamic acid	70.48	74.94	74.00
Glycine	383.70	393.94	367.72
Alanine	134.42	122.21	122.17
Valine	18.04	11.85	19.30
Methionine	5.11	0.00	0.00
Isoleucine	10.35	8.45	12.02
Leucine	20.41	20.90	30.68
Phenylalanine	11.36	10.70	12.12
Lysine	24.69	28.24	23.43
Histidine	7.10	6.43	5.20
Arginine	49.10	55.86	54.28
Proline	130.65	146.19	136.59

When the subunit compositions of FSCOL and BFCOL were further resolved by SDS-PAGE, Type I collagen, with several distinct band sizes corresponding to molecular weights of approximately 400 kDa and 250 kDa for γ- and β-chains respectively, and approximately 139 kDa and 129 kDa for α1- and α2-chains respectively, were visible after Coomassie Brilliant Blue staining for both FSCOL and BFCOL (Fig. 2b). In addition, the width ratio of the α1- and α2-chains for both FSCOL and BFCOL was found to be approximately 2:1, which is the typical ratio for Type I collagen [17, 18]. Also, the overall electrophoretic mobility of FSCOL and BFCOL were similar to the BVCOL, which was used as a positive control [32, 33]. Taken together, the results showed that Type I collagen could be successfully extracted from fish scales and bullfrog skin using the acid solubilisation method.

### Direct surface-initiated ATRP of PVDF surface for grafting of pHEMA brushes

**Fig. 3:**
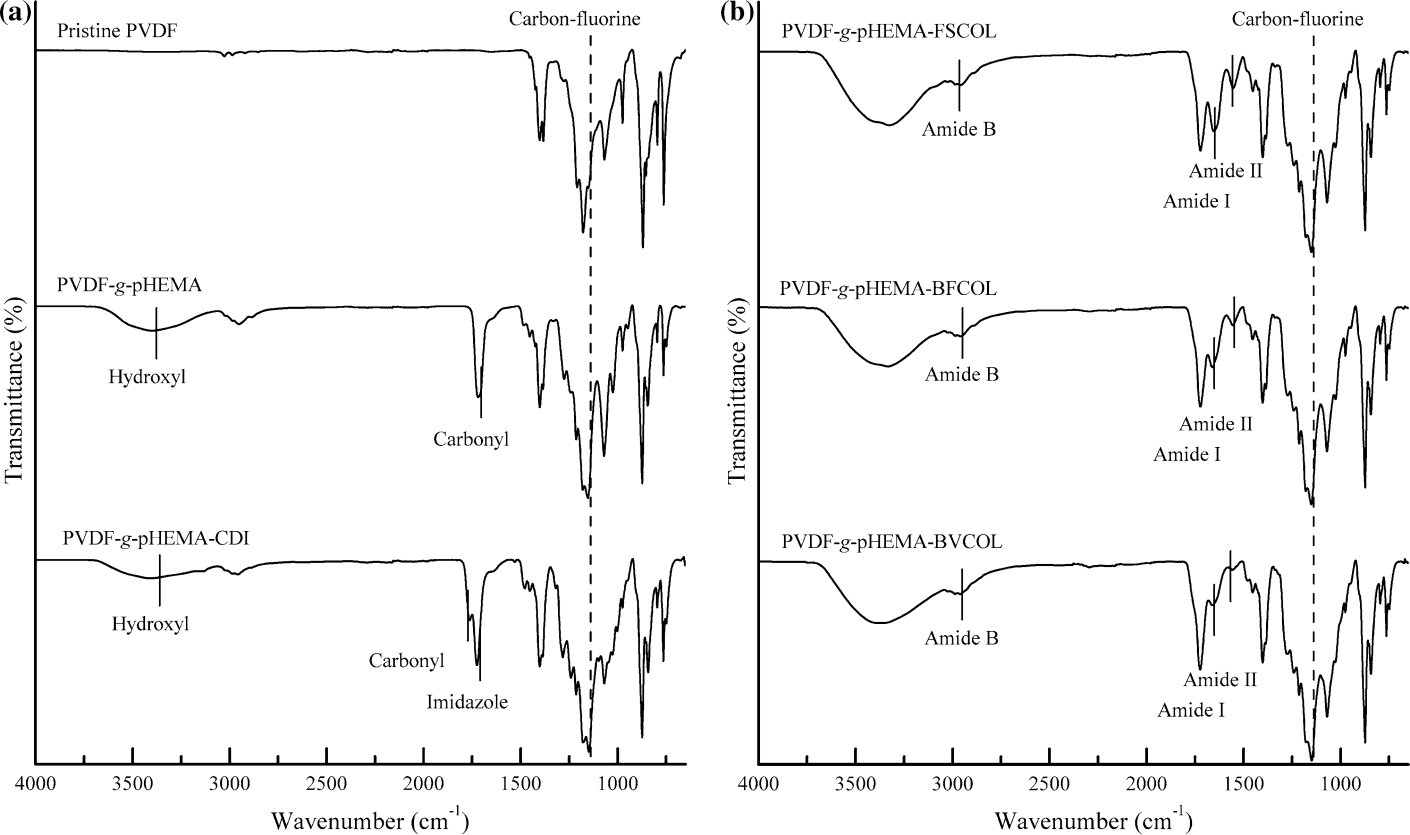
ATR-FTIR results after **a** Conjugation of pHEMA chains onto PVDF films followed by tethering of CDI biolinker onto the surface functionalised PVDF films and **b** Immobilisation of different sources of collagen onto the functionalised PVDF films. (*Dotted lines* Characteristic peaks for PVDF substrate; *Solid lines* Characteristic peaks resulted after each functionalisation ATRP step)

As shown schematically in Scheme 1, the reaction steps involved in the biofunctionalisation of PVDF include direct SI-ATRP from the pristine PVDF to graft pHEMA brushes, activation of hydroxyl groups on side chains of pHEMA brush with a CDI biolinker, and covalent conjugation of different sources of collagen. At each reaction step, chemical analysis by way of ATR-FTIR was carried out to identify peak changes. From the ATR-FTIR spectrum of pristine PVDF (Fig. 3a), the characteristic bands with wavenumber near 1177.91 cm^−1^ is attributed to –CF_2_ stretching vibration, other characteristic bands include the –C–H and –CH_2_ stretching with a wavenumber near 2986.22 and 1401.50 cm^−1^, respectively, the asymmetric stretching vibrations of the –CH_2_ groups at 3023 cm^−1^. Upon grafting of pHEMA, a broad absorption band near 3396.68 cm^−1^, attributable to terminal hydroxyl (-OH) group, was observed in the ATR-FTIR spectrum of PVDF-*g*-pHEMA. The appearance of an additional absorption peak at 1718.35 cm^−1^, attributable to the C=O stretching vibrations, indicates the presence of the ester carbonyl group of pHEMA [1]. Both characteristic peaks of pHEMA of –OH and C=O were absent in the spectrum of pristine PVDF, but present after the graft polymerisation reaction. Taken together, the ATR-FTIR spectra showed the successful grafting of the pHEMA onto the PVDF using the SI-ATRP method.

### Conjugation of collagen to the functionalised PVDF surface

In order to immobilise the extracted collagen to the functionalised PVDF films, a biofunctional linker, CDI, was introduced to activate the terminal –OH groups in side chains of pHEMA brushes. The resultant imidazole carbamate groups terminated PVDF-*g*-pHEMA films can be used to covalently conjugate the extracted collagen. The CDI modification of PVDF-*g*-pHEMA films resulted in the split of the absorption bands at 1762.53 cm^−1^ for the carbonyl groups of acrylate and 1724.85 cm^−1^ for the imidazole functionalities from the CDI compound (Fig. 3a) [34, 35]. The newly formed imide bond indicated the successful tethering of CDI onto the pHEMA-modified films. Also the decrease in the intensities of –OH stretching (near 3396.68 cm^−1^) was an indication of the conversion of hydroxyl group of pHEMA into the imidazole carbamate groups. Figure 3b shows the covalent immobilised collagen on the functionalised PVDF films. Additional characteristic absorption peaks of Type I collagen were observed on the ATR-FTIR spectrum showing the amide B (near 2958.86 cm^−1^, C–H stretching), amide I (near 1661.26 cm^−1^, C=O stretching) and amide II (near 1555.42 cm^−1^, C–N stretching and N–H bending) indicated successful immobilisation of collagen onto the functionalised PVDF films via SI-ATRP.

### Cellular studies

**Fig. 4:**
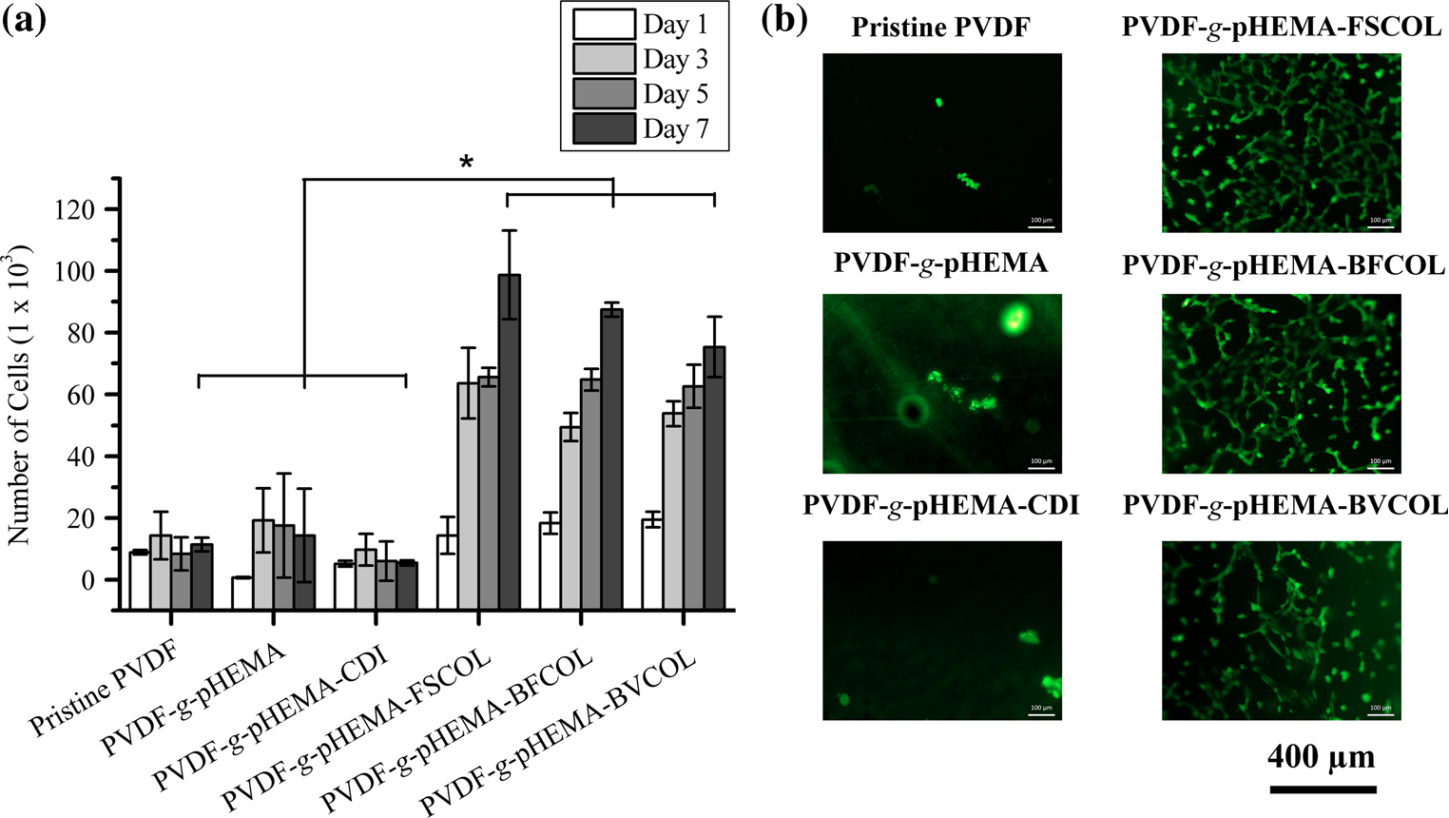
Cell proliferation results for the different ATRP-treated PVDF films using **a** PrestoBlue^®^ assay (*P < 0.05) and **b** FDA-stained viable HUVECs (*green* fluorescence) on Day 7 (Color figure online)

The cytocompatibility of the collagen-enriched PVDF films was evaluated by quantitatively measuring the cell adhesion and proliferation activity of HUVECs using the PrestoBlue^®^ assay over a 7 day period. It was observed that the hydrophobic pristine PVDF exhibited poor initial affinities for cell adhesion and hence could not support subsequent cell proliferation (Fig. 4a). For the pHEMA-modified PVDF films, even though PVDF-*g*-pHEMA and PVDF-*g*-pHEMA-CDI surfaces improved the hydrophilicity of the PVDF films due to the presence of hydrophilic polymer brushes on the hydrophobic PVDF surfaces and imidazole carbamate groups (Fig. S1), no significant improvements for cell attachment and cell proliferation profiles were observed due to the absence of biological motifs available to the cells. On the other hand, significantly higher (*P* < 0.05) cell attachment and growth was observed for the pHEMA-grafted PVDF films conjugated with the different sources of Type I collagen. As compared to the pristine PVDF films, the collagen-tethered films had at least 600 % more number of cells attached at day 7. However, no significant differences between the proliferation data of different species of collagen-enriched PVDF films could be observed, thus suggesting that both non-mammalian sources of collagen could be promising alternatives to the currently commercially available source.

In addition, observations from fluorescence staining of viable cells (Fig. 4b) were in agreement with the cell proliferation results, as the pristine PVDF film had the least number of cells attached at day 7. The PVDF-*g*-pHEMA and PVDF-*g*-pHEMA-CDI were observed to have low numbers of viable cells attached despite an improvement in surface hydrophilicity (Fig. S1). On the other hand, a confluent layer of cells was observed on the collagen-enriched PVDF surfaces conjugated with different sources of Type I collagen (PVDF-*g*-pHEMA-FSCOL, PVDF-*g*-pHEMA-BFCOL and PVDF-*g*-pHEMA-BVCOL). Overall, this confirmed that collagen-enrichment was important for promoting cell-material interactions for the endothelialisation of functionalised PVDF films.

### Gene expression studies

**Fig. 5:**
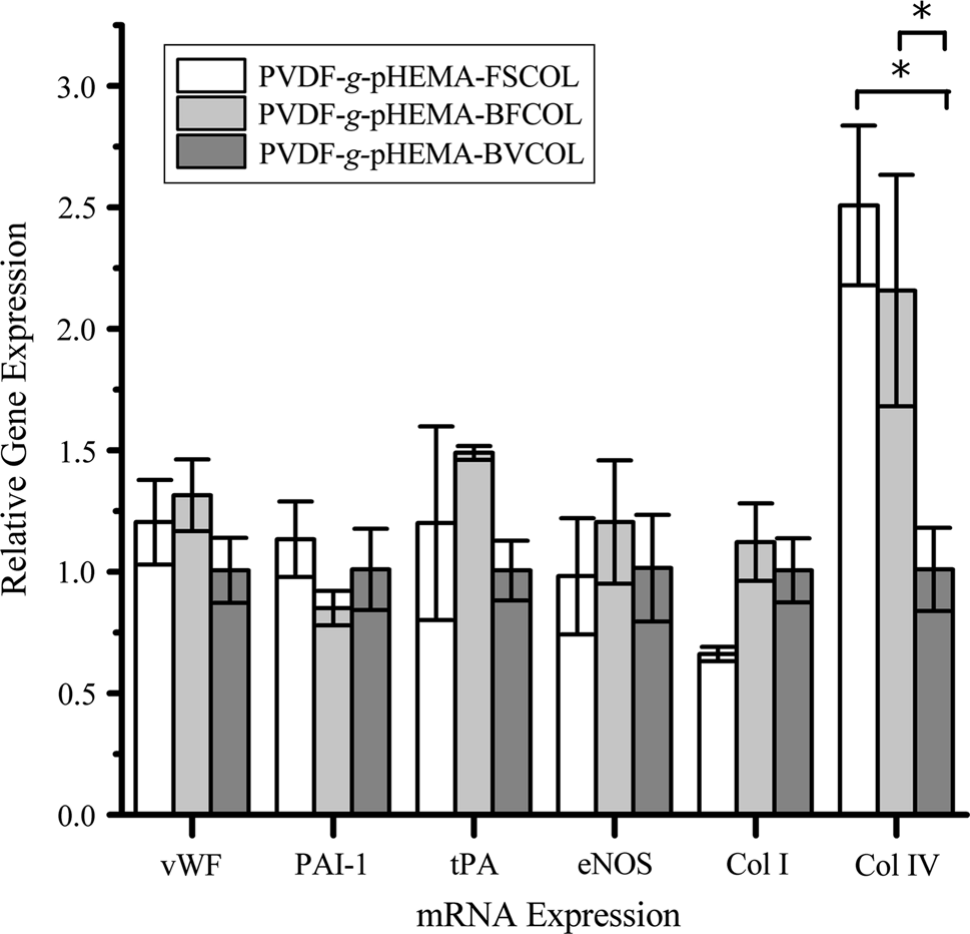
Relative mRNA expression of indicated genes as determined by qPCR when HUVECs were cultured on the different collagen-enriched substrates (*P < 0.05)

Figure 5 shows the gene expression levels for pro-thrombotic (vWF, PAI-1) and anti-thrombotic (tPA, eNOS) markers, which were measured from the cells growing collagen-enriched PVDF films. A lower PAI-1 but higher tPA mRNA expression level was observed for the cells growing on PVDF-*g*-pHEMA-BFCOL as compared to those on the PVDF-*g*-pHEMA-BVCOL substrates. Studies of PAI-1 have demonstrated that inhibiting PAI-1 could enhance thrombolysis in experimental animal models [36], while conversely, elevated levels of PAI-1 contributed to thrombus formation and venous occlusion [37]. PAI-1 is an important inhibitor of plasminogen activators, a class of key enzymes that potentiate fibrinolytic activity in the blood plasma, and of which tPA belongs to [38]. The secretion of tPA from HUVECs, together with its regulation in blood plasma levels by PAI-1, forms the important tPA/PAI-1 axis for maintaining basal anti-thrombotic activity in the blood plasma [39]. Overall, the tPA/PAI-1 ratios of FSCOL- and BFCOL-enriched PVDF films were higher than BVCOL. This suggests the non-mammalian collagens promoted a more anti-thrombogenic profile compared to BVCOL. Interestingly across the different sources of collagen, there were significantly higher (*P* < 0.05) expression levels of Type IV collagen in cells on FSCOL and BFCOL as compared to BVCOL. Type IV collagen is the main collagen type found in the subendothelial basement membrane of blood vessels and the presence of immobilised Type IV collagen is required for angiogenesis [40, 41]. Its requirement in angiogenesis has also been verified in three-dimensional organ cultures when it promoted neovessel outgrowth and stability [42]. Thus, FSCOL and BFCOL have seemed to be better at enhancing the pro-angiogenic potential of HUVECs than BVCOL.

### Platelet activation on different sources of collagen with endothelialised surface

**Fig. 6:**
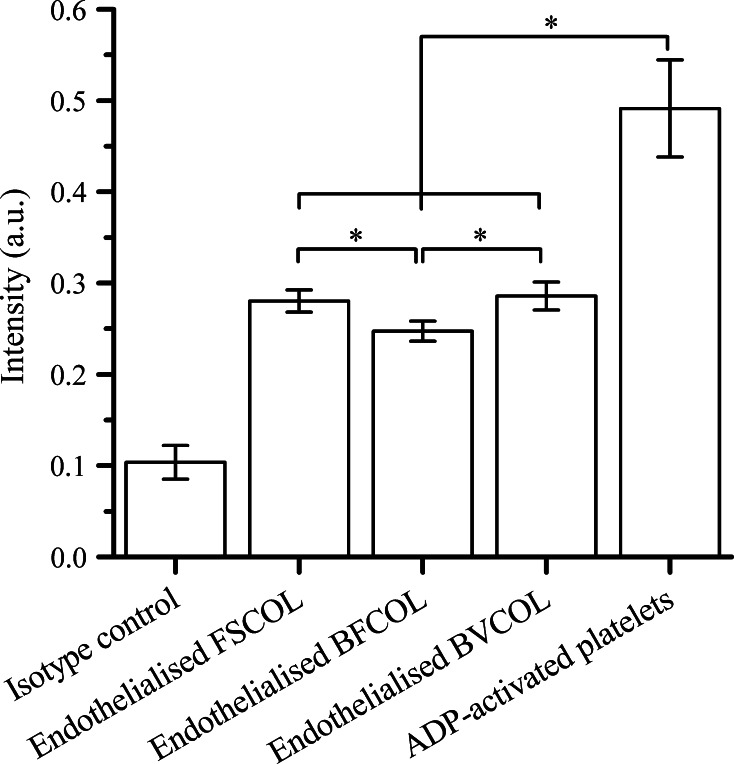
Expression of P-selectin (CD62P) on activated platelets plated on different endothelialised collagen substrates (*P < 0.05)

P-selectin is a cell adhesion molecule expressed by activated platelets and functions as a receptor for leukocyte binding [19]. Using a chemiluminescent immunoassay, P-selectin expression levels were quantitated on platelets attached to the endothelialised collagen surfaces, as an indication of the thrombogenicity of endothelial cells on the various collagen types. As shown in Fig. 6, on all the endothelialised collagen surfaces, the amount of activated platelets was significantly lower (*P* < 0.05) than the ADP-activated platelets. ADP is a known agonist for platelet activation and aggregation [43], and in our study ADP-activated platelets serve as a suitable benchmark for assessment of platelet activation by the collagen surfaces. The difference between the platelet activation amongst the three different sources of collagen was small, but statistically significant (*P* < 0.05), which is an indication that BFCOL had a lower risk of thrombogenesis as compared to other sources of collagen. Overall, significantly lower amounts of activated platelets were observed on these different sources of collagen when compared to ADP activation, suggesting that hemocompatibility was better on the endothelialised collagen surfaces.

### ICAM-1 and VCAM-1 expression of endothelial cells cultured on different sources of collagen

**Fig. 7:**
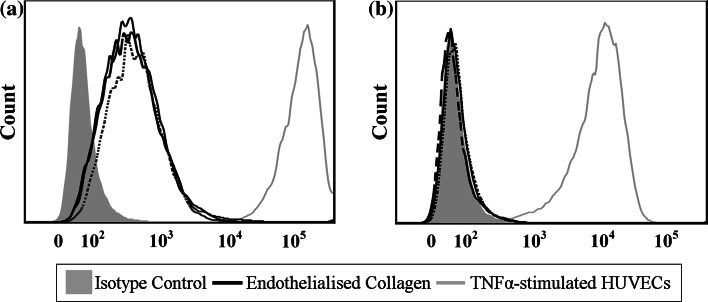
Representative FACS analysis of HUVECs expressing **a** ICAM-1 expression and **b** VCAM-1 expression, when cultured on different endothelialised collagen surfaces (*Solid line* FSCOL; *Dashed line* BFCOL and *Dotted line* BVCOL)

ICAM-1 and VCAM-1 are the leukocyte adhesion molecules on the surface of HUVECs, which also serve as markers of endothelial cell activation and pro-inflammatory responses as an indication of the inflammatory reaction of the material-tissue interface [24]. In this study, the expression of both molecules was quantitated by flow cytometry as a measure of the inflammatory state of HUVECs on different sources of collagen (Fig. 7) [44]. Overall, HUVECs cultured on endothelialised collagen surfaces had lower cell surface ICAM-1 levels than TNFα-stimulated HUVECs. From the ICAM-1 intensity histograms, HUVECs on FSCOL- and BFCOL-enriched surfaces had lower peak intensities with median fluorescence intensities (MFI) of 344 and 340 respectively, compared to the BVCOL (MFI = 377). In general, the cells do not typically express VCAM-1 unless they are stimulated with cytokines [45]. The HUVECs on all the collagen-enriched surfaces remained negative for cell surface VCAM-1 expression, indicating that they were not activated to exhibit a pro-inflammatory state. As expected, TNFα-stimulated HUVECs induced a highly upregulated expression of VCAM-1. Thus, from the flow cytometry data, all collagen sources tested in this study did not increase the expression of leukocyte adhesion molecules of the HUVECs cultured on them, and maintained a non-inflammatory state in these cells.

## Discussion

Prior to the collagen extraction process, SEM analysis (Fig. 1) showed the presence of collagen fibres in both fish scale and bullfrog skin. Following that, the collagen was extracted from the fish scales and bullfrog skin using acid solubilisation method and characterised by chemical analysis and SDS-PAGE. Although the collagen fibres from both fish scale and bullfrog skin were observed to have different fibre orientations and fibre diameters, they showed similar profiles when subjected to ATR-FTIR analysis and SDS-PAGE. In general, all the ATR-FTIR spectra for the extracted collagen showed similar profiles, where the presence of typical secondary protein structures of collagen such as amides A, B, I, II and III were found, which confirmed the successful isolation of collagen from the waste materials (Fig. 2a). Further corroboration that the acid solubilisation method used in this study was able to preserve the quality of the collagen with the presence of 1450 cm^−1^ peak, which is an indication of the presence of the triple helical structure in collagen. From the ATR-FTIR results, both extracted FSCOL and BFCOL had similar absorbance peak intensity ratios of amide III to 1450 cm^−1^, as that of BVCOL (around 1.0), which is an indication that the acid solubilisation method maintained the similar fibril-forming ability of the ASC as well as the structural integrity and stability as compared to the commercially available source [29]. Subsequently, SDS-PAGE analysis (Fig. 2b) showed similar profiles for the collagen isolated from both fish scales and bullfrog skin to that of the BVCOL. The presence of two main α-chains, α1- and α2, is an indication that the major component of the extracted collagen is that of Type I collagen. This is similar to the BVCOL Type I collagen that is often used as a material in the engineering of vascular constructs as it is the most abundant collagen type found in vascular tissue and has the primary role of providing biomechanical support [46].

Once the extracted collagen has been characterised and confirmed to be that of Type I collagen, it was then conjugated onto pHEMA-modified PVDF films via the SI-ATRP process. As the secondary fluorine atoms of the PVDF backbone can act as halogenated sites to initiate graft polymerisation, pretreatment of PVDF films for surface activation was not required in this case. In general, ATRP is known for its controlled radical polymerisation approach where the grafting amount of the pHEMA brushes is directly proportional to the ATRP reaction time (Fig. S2). In this study, conjugation of Type I collagen onto the surfaces of functionalised PVDF films was confirmed by ATR-FTIR analysis (Fig. 3) following each modification step. The robust covalent binding of collagen onto the PVDF film surface using the SI-ATRP method ensured that the collagen coating, and hence HUVECs, remained on the substrates over time as compared to physically-coated collagen on PVDF films (Figs. S3 and S4). Although the denaturation temperature (T_d_) of the non-mammalian sources of collagen was lower compared to BVCOL (Fig. S5), these non-mammalian sources of collagen did not easily undergo dissolution, thus allowing for the preservation of the biocompatibility of the PVDF films. In fact, the collagen-enriched PVDF films were able to support the proliferation of the HUVECs until confluency on day 7 due to this robust covalent binding process. The presence of collagen is important, as the positively charged *N*-containing groups of collagen at physiological pH promote interactions with negatively charged cells [47]. This was further confirmed in our study when we compared surface functionalised PVDF films with and without conjugation of collagen (Fig. 4), and also from earlier studies where we showed that an improvement in surface hydrophilicity did not necessarily lead to improved cell attachment and proliferation if collagen was absent (Fig. S1). In general, collagen-enrichment resulted in a significant improvement (*P* < 0.05) in cell proliferation, but amongst different sources of collagen-enriched PVDF films, no significant difference was observed. In fact, even though BFCOL had higher amounts of lysine (Table 2), which can act as the primary amine for intra- and inter-molecular crosslinking reactions to facilitate the conjugation of collagen onto the PVDF films [48], no significant improvement in cell proliferation results were observed for the BFCOL-enriched films. Hence, any possible increase or reduction in the availability of crosslinking sites for surface conjugation did not appear to exert any significant effect on cellular behaviour. Since the ATRP method is a controlled radical polymerisation approach with well-defined amounts of chain growth, we postulate that regardless of the amount of lysine residue, the well-grafted CDI chains led to sufficient reactive groups for thorough surface coverage, thus ensuring that every single collagen chain was in direct conjugation with the surface. In addition, the ATR-FTIR analysis and SDS-PAGE of the different sources of collagen showed that they were of the same type (i.e. Type I collagen), with similar triple helical conformations, and therefore interacted with the collagen-binding integrins (α1β1, α2β1 and α11β1) of the HUVECs to a similar extent [49]. Overall, the conjugation of Type I collagen onto PVDF films improved the cell-material interaction, resulting in good cell proliferation regardless of source. This suggests that collagen derived from food processing waste, fish scales and bullfrog skin, could be promising alternative sources of collagen. With enhanced endothelialisation and thus improved biocompatibility of collagen-enriched PVDF films, further characterisations were carried out to assess the hemocompatibility of the different sources of collagen in terms of the phenotypic expression of HUVECs cultured on the collagen-enriched PVDF films.

Based on the mRNA expression levels (Fig. 5), HUVECs cultured on FSCOL- and BFCOL-enriched PVDF films exhibited significantly higher (*P* < 0.05) expression levels of Type IV collagen compared to BVCOL-enriched PVDF films. This suggests that HUVECs cultured on FSCOL- and BFCOL-enriched PVDF films exhibited a higher propensity to be pro-angiogenic, as Type IV collagen is a constituent of the basement membrane deposited by angiogenic endothelial cells [50]. In addition, Type I collagen is known to promote angiogenesis, since the fibrillar structure of Type I collagen could promote ligation or clustering of the integrin receptor of HUVECs, which is important for the activation of signalling pathways for angiogenesis [51]. Taken together, PVDF films conjugated with FSCOL and BFCOL exhibited improved endothelialisation and pro-angiogenic properties. In addition, higher tPA/PAI-1 ratios were detected in HUVECs cultured on FSCOL- and BFCOL-enriched PVDF films, as compared to BVCOL-enriched films. Since PAI-1 is a pro-thrombotic marker and tPA is an anti-thrombotic marker, this suggests the non-mammalian collagens promoted a more anti-thrombogenic profile.

This improved anti-thrombogenic property of FSCOL and BFCOL was further confirmed from the platelet activation studies carried out on the endothelialised substrates (Fig. 6). In general, platelet activation is considered an important criterion in the assessment of the hemocompatibility of biomaterials, since the activation of the attached platelet on the biomaterials’ surface could lead to the aggregation and the formation of thrombus [22]. Also, collagen receptors have been identified on platelets, most notably α2β1 integrin and the immunoglobulin superfamily GPVI, which is why the direct interaction of collagen with platelets has been known to trigger the activation of thrombogenic initiation [19]. Therefore, the endothelialisation of the collagen surfaces is an important step in the reduction of collagen-induced thrombogenicity. In the current study, the amount of P-selectin expression of the platelets was investigated, since it is one of the biomarkers found to be increased on the platelet surface following activation [52]. The extent of platelet activation could be determined by comparing the P-selectin expression on platelets attached to the endothelialised collagen surfaces with the ADP-activated platelets [19, 22]. From the results (Fig. 6), all the platelets in contact with the endothelialised collagen surfaces did indeed have significantly lower (*P* < 0.05) platelet activation as compared to ADP-activated platelets. Furthermore, the lowest amount of activated platelets was observed on the endothelialised BFCOL surfaces. In addition, the plasma recalcification assay was performed on different sources of endothelialised collagen surfaces by incubating the surfaces with platelet poor plasma, followed by measuring fibrin clot initiation times when the plasma was recalcified with calcium chloride [53]. The clot initiation times for FSCOL, BFCOL and BVCOL were 17.5 ± 3.6, 16.9 ± 1.1 and 16.1 ± 3.5 min respectively, which is significantly longer (*P* < 0.05) than the timings of TCPs control and pristine PVDF with timings of 9.4 ± 0.7 and 6.0 ± 0.5 min, respectively (Fig. S6). Similar to the results from the platelet activation study, the endothelialised collagen surfaces had a significantly longer (*P* < 0.05) clot formation time as compared to TCPs control and pristine PVDF. In addition, the non-mammalian sources of collagen had slightly prolonged clot formation times as compared to BVCOL. Taken together, the endothelialised collagen surfaces demonstrated better anti-coagulation responses, which is a phenomenon that is also reported by others [54, 55].

Lastly, the material-induced inflammatory response of HUVECs in contact with different sources of collagen was studied by investigating level of activation of HUVECs cultured on the collagen. The activation of HUVECs is typically associated to the initial step of inflammation, caused by the increased infiltration of leukocytes into the inflamed tissue, which is mediated by leukocyte adhesion molecules such as ICAM-1 and VCAM-1. Thus, the activation of HUVECs is commonly used to investigate inflammatory reactions [25]. From the results (Fig. 7), the cells on collagen-enriched surfaces all have low constitutive expression levels of ICAM-1 with little or no expression levels of VCAM-1. These values were compared to TNFα-stimulated HUVEC controls, since TNFα is a pro-inflammatory cytokine known to increase the expression of cell adhesion molecules [44]. Taken together, the expression profiles of the HUVECs on all the collagen-enriched surfaces used in this study is in line with that of inactivated HUVECs in culture (i.e. low constitutive expression of ICAM-1, no expression of VCAM-1) [23]. Overall, it can be deduced that the non-mammalian sources of collagen, similar to the BVCOL, did not trigger a pro-inflammatory phenotype in the HUVECs.

## Conclusion

Type I collagen was successfully extracted from fish scales and bullfrog skin and chemically conjugated onto PVDF films using the direct SI-ATRP approach. The acid solubilisation method used in this study led to the preservation of the quality of the extracted collagen, since FSCOL and BFCOL both had similar triple helical confirmation to that of commercially available Type I BVCOL. The collagen-enriched PVDF films significantly enhanced (*P* < 0.05) cell-biomaterial interactions by improving the cell attachment and cell proliferation, which then led to a better coverage of HUVECs on the collagen-enriched PVDF films. As compared to BVCOL, the non-mammalian sources of collagen led to endothelialised surfaces with higher expression levels of Type IV collagen by HUVECs and comparable anti-thrombogenic properties, thus making them more suitable candidates for blood contacting applications. In addition, the low expression levels of ICAM-1 and VCAM-1 indicated that these non-mammalian sources of collagen did not elicit pro-inflammatory phenotypes in the cells. Taken together, our study not only has highlighted the feasibility of using FSCOL and BFCOL as cost effective substitutes for BVCOL, but has also demonstrated an effective chemical functionalisation scheme for PVDF substrates via SI-ATRP process. The conversion of the abundantly available food processing wastes, fish scales and bullfrog skin, into collagen is a demonstration of an effective waste-to-resource management to utilise the underexploited biological resources. These sources of non-mammalian collagen can potentially be used as alternative resource resilient biomaterials for tissue engineering applications.

## Electronic supplementary material

Supplementary material 1 (DOCX 640 kb)
