# Supplementary material for: Surface modification of PVDF using non-mammalian sources of collagen for enhancement of endothelial cell functionality
Source: J Mater Sci Mater Med. 2016 Jan 12;27:45. doi: 10.1007/s10856-015-5651-8 (PMC4710638; doi:10.1007/s10856-015-5651-8)
Supplement: Supplementary file 1 — Supplementary material 1 (DOCX 640 kb) [file 10856_2015_5651_MOESM1_ESM.docx]

Supplementary Data

**Surface modification of PVDF using non-mammalian sources of collagen for enhancement of endothelial cell functionality**

Jun Kit Wang ⋅ Gordon Minru Xiong ⋅ Baiwen Luo ⋅ Chee Chong Choo ⋅ Shaojun Yuan ⋅ Nguan Soon Tan ⋅ Cleo Choong

J. K. Wang

Residues and Resource Reclamation Centre (R3C), Nanyang Environment and Water Research Institute (NEWRI), Nanyang Technological University, 1 Cleantech Loop, Singapore 637141

J. K. Wang

Interdisciplinary Graduate School, Nanyang Technological University, 50 Nanyang Avenue, Singapore 639798

J.K. Wang *⋅* G. Xiong *⋅* B. Luo *⋅* C. Choong*

School of Materials Science and Engineering, Nanyang Technological University, 50 Nanyang Avenue, Singapore 639798

Telephone: +65 6513 8166

Email: cleochoong@ntu.edu.sg

C. C. Choo *⋅* N. S. Tan

School of Biological Sciences, Nanyang Technological University, 60 Nanyang Avenue, Singapore 637551

S. Yuan

College of Chemical Engineering, Sichuan University, 19 Wangjiang Road, Wuhou, Chengdu, Sichuan, China

N. S. Tan

Institute of Molecular and Cell Biology, 61 Biopolis Drive, Proteos, A*STAR, Singapore 138673

N.S. Tan *⋅* C. Choong

KK Research Centre, KK Women’s and Children Hospital, 100 Bukit Timah Road, Singapore 229899


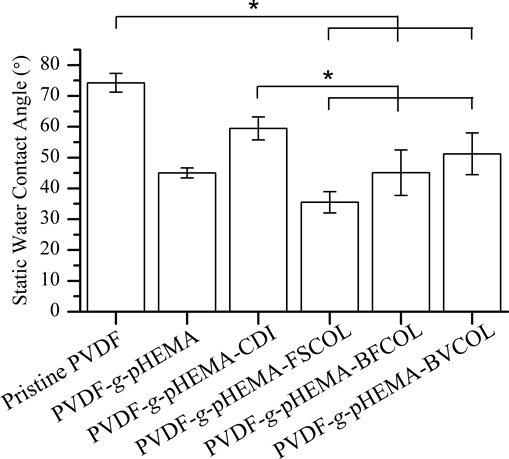


**Fig. S1.** Static water contact angle values of the PVDF films at different stages of the ATRP process. Overall, collagen-enrichment of PVDF led to a significant improvement (**p*<0.05) in surface hydrophilicity


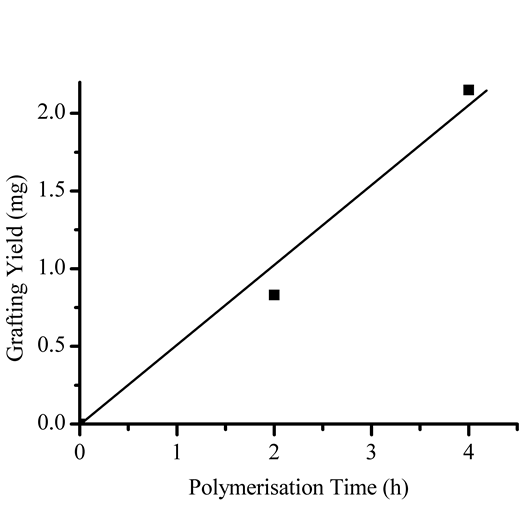


**Fig. S2.** Grafting yield from the ATRP process showing a linear relationship with respect to time


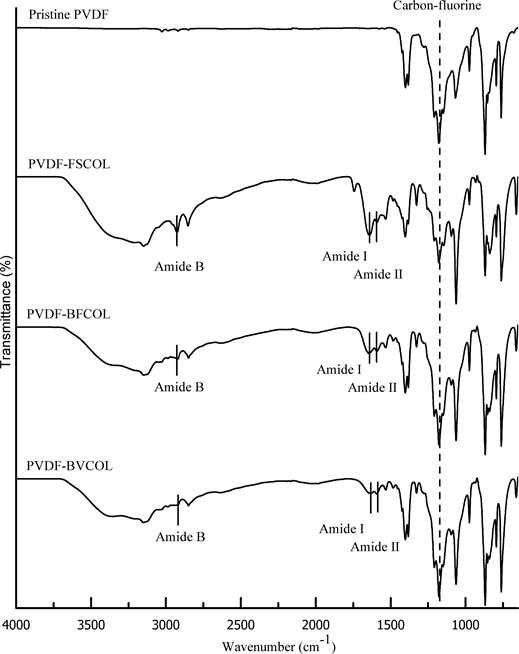


**Fig. S3.** ATR-FTIR results showing the presence of collagen physisorption on PVDF films. (Dotted lines: Characteristic peaks of PVDF substrate; Solid lines: Characteristic amide peaks of collagen)


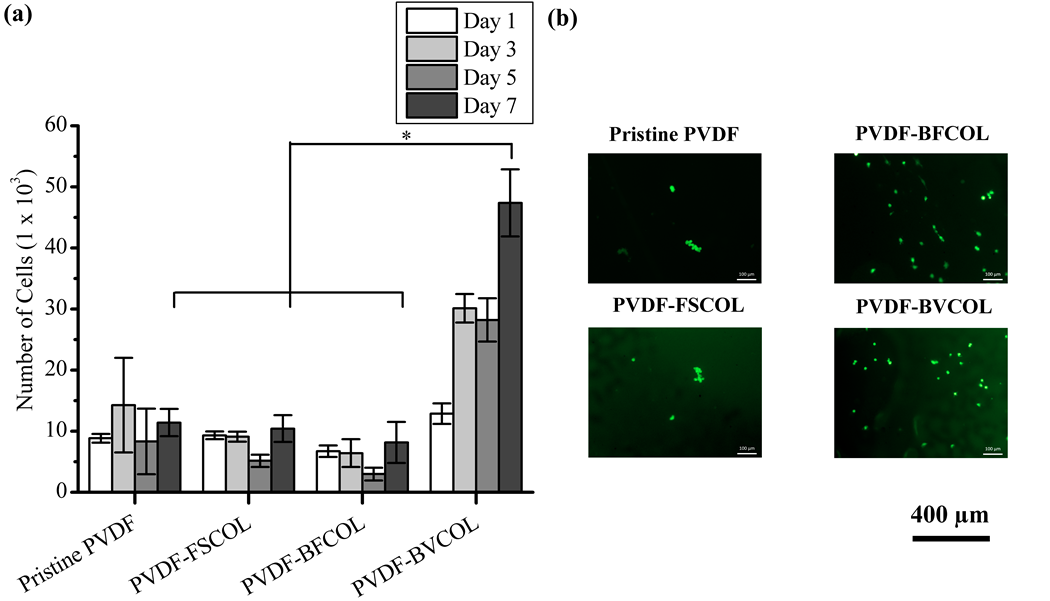


**Fig. S4.** Cell proliferation results for the collagen physisorption on PVDF films using (a) PrestoBlue® assay (**p*<0.05) and (b) FDA-stained viable HUVECs (green fluorescence) on Day 7


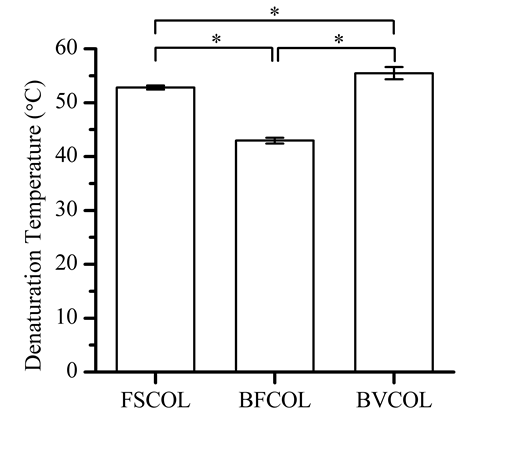


**Fig. S5.** Denaturation temperatures of different sources of collagen as measured using differential scanning calorimetry (DSC) with a temperature range of 25 – 80 °C and heated at a rate of 2.0 °C/min (**p*<0.05).


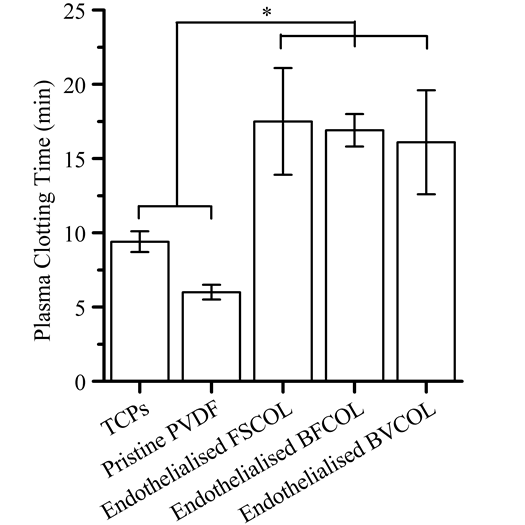


**Fig. S6.** Results from the plasma recalcification assay showing the clot initiation time of the recalcified platelet-poor plasma on different types of material surfaces. The plasma clotting time was taken as the point of spike formation under the optical density reading of 405 nm (**p*<0.05).
